# Dehydration, Rehydration and Thermal Treatment: Effect on Bioactive Compounds of Red Seaweeds *Porphyra umbilicalis* and *Porphyra linearis*

**DOI:** 10.3390/md22040166

**Published:** 2024-04-09

**Authors:** Carla Pires, Maria Sapatinha, Rogério Mendes, Narcisa M. Bandarra, Amparo Gonçalves

**Affiliations:** 1 Division of Aquaculture, Upgrading and Biospropecting (DivAV), Department for the Sea and Marine Resources, Portuguese Institute for the Sea and Atmosphere (IPMA), Av. Dr. Alfredo Magalhães Ramalho 6, 1495-165 Lisbon, Portugal; maria.sapatinha@ipma.pt (M.S.); rogerio@ipma.pt (R.M.); narcisa@ipma.pt (N.M.B.); amparo@ipma.pt (A.G.); 2 Interdisciplinary Center of Marine and Environmental Research (CIIMAR), University of Porto, Terminal de Cruzeiros de Leixões, Av. General Norton de Matos s/n, 4450-208 Matosinhos, Portugal

**Keywords:** antioxidant and anti-Alzheimer’s activities, carotenoids, flavonoids compounds, phycobiliproteins, total phenolic compounds

## Abstract

The nutritional and bioactive value of seaweeds is widely recognized, making them a valuable food source. To use seaweeds as food, drying and thermal treatments are required, but these treatments may have a negative impact on valuable bioactive compounds. In this study, the effects of dehydration, rehydration, and thermal treatment on the bioactive compounds (carotenoids, phycobiliproteins, total phenolic content (TPC), total flavonoids content (TFC)), antioxidant (ABTS and DPPH radical scavenging activities) and anti-Alzheimer’s (Acetylcholinesterase (AchE) inhibitory activities, and color properties of *Porphyra umbilicalis* and *Porphyra linearis* seaweeds were evaluated. The results revealed significant reductions in carotenoids, TPC, TFC, and antioxidant activities after the seaweeds’ processing, with differences observed between species. Thermal treatment led to the most pronounced reductions in bioactive compound contents and antioxidant activity. AchE inhibitory activity remained relatively high in all samples, with *P. umbilicalis* showing higher activity than *P. linearis.* Changes in color (ΔE) were significant after seaweeds’ dehydration, rehydration and thermal treatment, especially in *P. umbilicalis.* Overall, optimizing processing methods is crucial for preserving the bioactive compounds and biological activities of seaweeds, thus maximizing their potential as sustainable and nutritious food sources or as nutraceutical ingredients.

## 1. Introduction

Due to the projected growth of the world population to 10 billion over the next thirty years, a 70% boost in food production will be necessary. As a result, finding novel food sources is crucial, and seaweeds are considered to be one of the most promising reserves of sustainable food. Algae production is a growing industry worldwide, with a focus on developing sustainable and environmentally friendly methods for producing algae-based products. According to the Food and Agriculture Organization (FAO), global algae production reached approximately 36 million metric tons in 2019, with the majority of production (97.4%) taking place in Asia [1]. The nutritional value of seaweeds is widely recognized: they are rich in dietary fiber and low in fat (but including the beneficial omega-3 fatty acids), contain all essential amino acids, are abundant in various vitamins, and are an exceptional source of minerals and other essential nutrients, making them a valuable food source [2]. They are also an interesting and promising source of bioactive compounds with health-promoting benefits, such as pigments, polysaccharides, phenolics, proteins, and lipids. Carotenoids, the most common pigments found in nature, are present in all types of algae, and red seaweed species are particularly abundant in pigments such as α- and β-carotene, lutein, and zeaxanthin [3].

Red seaweeds are also known to contain high concentrations of phycobiliproteins, which are responsible for the characteristic red or purple coloration of these algae. The phycobiliproteins found in red seaweeds include R-phycocyanin, R-phycoerythrin, and allophycocyanin [4]. Carotenoids and phycobiliproteins have potential applications as a source of natural food colorants that can be applied to many products. Seaweeds contain various phenolic compounds like phlorotannins, bromophenols, flavonoids, phenolic terpenoids, and mycosporine-like amino acids that have beneficial effects on health [5]. In red seaweeds, the most significant phenolic compounds are bromophenols, flavonoids, phenolic acids, phenolic terpenoids, and mycosporine-like amino acids [6]. The properties of these bioactive compounds include antioxidant, anti-inflammatory, antimicrobial, anti-cancer, anti-obesity, anti-hypertensive, anti-diabetic and anti-Alzheimer’s activities [7].

Typically, fresh seaweed contains a high amount of moisture, reaching up to 95% depending on the species. As a result, the food industry often dehydrates seaweed before incorporating it into their products in order to enhance its shelf life, prevent decomposition, and facilitate the extraction of certain chemical compounds [8]. However, phenolic compounds, proteins, lipids, and other bioactive compounds can be negatively impacted by the drying process, thereby diminishing seaweed’s overall quality. Several drying methods (sun-drying, oven-drying, freeze-drying, and vacuum-drying, and more recently, microwave-drying) have been utilized for the drying of seaweeds [9]. However, to choose the most appropriate method, it is essential to take into account factors such as cost, energy usage, efficiency, and impact on the quality of the final product [10]. Additionally, cooking methods (rehydration, cooking) also have an impact on the bioactive compounds of seaweeds. Pigments are some of the components most affected by these methods, which can induce changes in the structure and stability of these compounds, leading to alterations in the color of seaweeds. So, it is essential to evaluate how these processing steps impact and modify seaweed pigments’ color and bioactivities, which are so valued for their beneficial nutritional and health effects.

Thus, the purpose of this study was to evaluate the effect of dehydration, rehydration and thermal treatment on the contents of bioactive compounds and antioxidant and anti-Alzheimer activities of the wild red seaweed species *Porphyra umbilicalis* and *Porphyra linearis*. The main focus was to test the lowest temperatures in order to preserve the bioactive benefits deriving from red seaweeds’ consumption. This work was carried out within a wider study aiming to evaluate the effect of those factors on the overall quality of wild red seaweeds, including sensory properties and nutritional value (the other manuscript is in preparation).

## 2. Results and Discussion

### 2.1. Carotenoid Content

Carotenoids are important pigments found in red algae, contributing to their distinctive colors and playing essential roles in various biological processes such as the photosynthetic process. Carotenoids find applications in the food and cosmetic industries [11].

The neurosporene, α-Carotene, lutein, zeaxanthin, fucoxanthin, β-Carotene and astaxanthin contents of the different seaweed samples are listed in Table 1. In general, fresh seaweeds presented higher levels of carotenoids, and as can be seen, there was a loss of carotenoid content after dehydration, rehydration, and thermal treatment. In all samples, fucoxanthin was the pigment that showed the highest levels.

Contrary to *P. umbilicalis*, the levels of carotenoids of *P. linearis* decreased with rehydration but increased when the heat treatment was applied afterwards. In *P. linearis* samples, the highest percentage reduction in carotenoid levels occurred with rehydration (approximately 57%). On the contrary, in the case of *P. umbilicalis* samples, the highest percentage of carotenoids reduction occurred with heat treatment (around 60%). Furthermore, the levels of neurosporene, α-Carotene, lutein, zeaxanthin, fucoxanthin, β-Carotene, and astaxanthin in *P. umbilicalis* and *P. linearis* samples were quite similar.

The results obtained for lutein levels in *P. linearis* were in accordance with those reported by Pina et al. (2014) and Amorim-Carrilho et al. (2014), who observed higher levels of this carotenoid in boiled seaweeds than in rehydrated samples of *Chondrus crispus* and *Himanthalia elongata* [12,13]. Amorim et al. (2012) also found high levels of lutein in boiled *Undaria pinnatifida* and *Laminaria* sp. seaweeds [14]. The same behavior was reported by these authors for the levels of fucoxanthin and β-carotene. The chlorophyll and carotenoid contents of *Ulva lactuca* collected from the Tabuk coast (Saudi Arabia) were greater after culinary treatment (boiling, 5 min) in comparison with raw algae [15]. According to these authors, this increase after thermal treatment may be related to the higher availability of these compounds for extraction. 

### 2.2. Phycobiliproteins

Phycobiliproteins play a significant role in the photosynthetic machinery of red algae. These pigments are crucial for capturing light energy and facilitating photosynthesis. Their unique light-harvesting properties have ecological significance and find applications in various industrial fields [11].

The results obtained for phycoerythrin, phycoerythrocyanin, phycocyanin, allophycocyanin, and allophycocyanin-β are shown in Table 2. The most abundant phycobiliprotein in all samples was phycoerythrin, followed by phycoerythrocyanin and phycocyanin. In general, dehydration and rehydration of *Porphyra* samples did not cause a significant decrease in the levels of phycoerythrin, phycoerythrocyanin, and phycocyanin, but the heat treatment led to a significant reduction in the levels of these phycobiliproteins. In the case of allophycocyanin, dehydrated samples exhibited higher levels than fresh samples, and rehydration and thermal treatment caused a significant decrease in this phycobiliprotein in both *Porphyra* samples. In general, the levels of allophycocyanin-β in dehydrated, rehydrated, and thermally treated *P. linearis* samples were higher than in fresh samples. However, no significant differences were observed in the content of allophycocyanin-β between samples. Except for the fresh samples, the concentrations of phycoerythrin, phycoerythrocyanin, phycocyanin, allophycocyanin, and allophycocyanin-β were lower in *P. umbilicalis* compared to *P. linearis* samples.

The significant loss of phycoerythrin, phycoerythrocyanin, and phycocyanin with thermal heating could be the result of the denaturation of these proteins at 90 °C. As stated by Pina et al. (2014), phycoerythrin denatures at temperatures above 45 °C at pH 7.0 and phycocyanin at approximately 60–65 °C [12].

### 2.3. TPC and TFC

Phenolic compounds, identified as secondary metabolites, are present in seaweeds. The phenolic compounds found in seaweeds encompass a broad spectrum, and this category also includes flavonoids—a substantial group within the realm of secondary metabolites.

**Figure 1:**
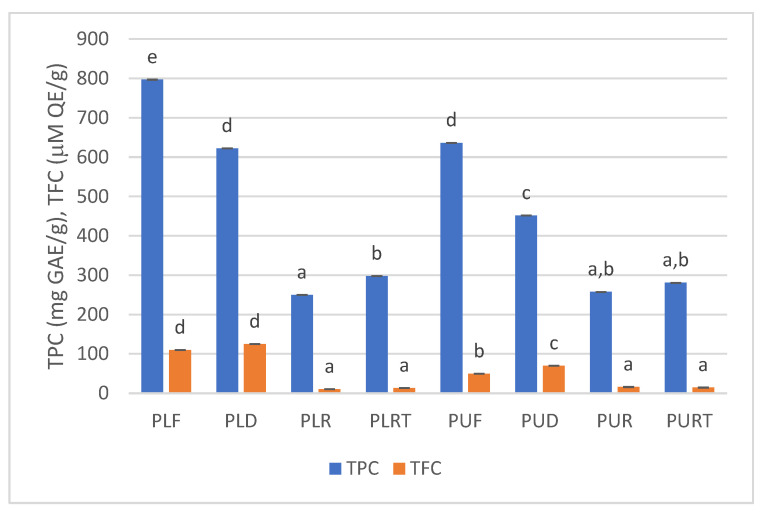
Total phenolic and flavonoids content of fresh (F), dehydrated (D), rehydrated (R) and thermally processed (RT) *P. linearis* (PL) and *P. umbilicalis* (PU) samples. Different letters indicate significant differences (*p* < 0.05) between samples for TPC and TFC.

Figure 1 shows the effect of dehydration, rehydration, and thermal treatment on the total phenolic and flavonoid content of *Porphyra* samples. As can be seen, the highest levels of TPC were observed in fresh samples (PLF = 797 mg GAE/g dw, PUF = 636 mg GAE/g dw), and dehydration caused a decrease in TPC around 29 and 22% for *P. linearis* and *P. umbilicalis*, respectively. Moreover, the TPC levels in both fresh and dehydrated samples of *P. linearis* were significantly higher than those of *P. umbilicalis*.

The rehydration and thermal treatment of *Porphyra* seaweeds caused a significant decrease in the TPC content, with a more pronounced reduction observed in rehydrated samples (ca 69% in *P. linearis* and 60% in *P. umbilicalis)* compared to those subjected to high temperatures (ca 63% in *P. linearis* and 56% in *P. umbilicalis*).

Gupta et al. (2011) also observed in a study with *H. elongata* that drying at lower temperatures (25 and 30 °C) resulted in a decrease in TPC. As pointed out by these authors, the lower drying temperatures probably did not inactivate the oxidative enzymes, which may have resulted in some oxidation of phenolic compounds and in lower TPC. The decrease in TPC during drying can also be attributed to the binding of polyphenols with proteins, for example, or to the alterations in the chemical structure of polyphenols, which cannot be extracted or determined by available methods [16].

Several studies have also reported the negative effect of different cooking methods on phenolic compounds. Jiang et al. (2022) investigated the effect of some cooking methods (blanching, steaming and boiling) on the TPC of *U. pinnatifida* and found that all of them reduced the TPC [17]. As referred by these authors, high-temperature oxidation, leaching during cooking, and dissolution of phenolic compounds in hot water may lead to the loss of polyphenols. However, Susanto et al. (2017) observed that heat treatments, i.e., blanching, boiling, steaming and sterilizing, did not affect the TPC content of *Sargassum ilicifolium* [18]. Rajauria et al. (2010) found that temperatures of 100, 110, and 121 °C caused a significant decrease in the TPC levels in samples of *H. elongata*, *Laminaria saccharina*, and *Laminaria digitata*. However, these authors also reported that samples heated to 85 °C and 95 °C exhibited higher levels compared to fresh samples [19]. 

The effects of dehydration, rehydration, and thermal treatment on total flavonoids content (TFC) are shown in Figure 1. The highest levels of TFC were detected in dehydrated samples (PLD = 125 µM QE/g dw and PUD = 70 µM QE/g dw), and in the case of *P. umbilicalis*, the dehydration significantly increased (ca 41%) the TFC.

The rehydration and heat treatment caused a significant decrease (ca 90% in *P. linearis* and 70% in *P. umbilicalis*) in the TFC content of both seaweeds.

Similar reductions in TFC levels were observed when *H. elongata* was dried at 25 °C (49%) and 40 °C (30%) [16]. Badmus et al. (2019) evaluated the effect of oven-drying (40 and 60 °C, 48 h), freeze-drying (−60 °C, 5 days), and microwave-drying on TFC of edible brown seaweeds, and in all the studied species, no consistent TFC patterns were observed [8]. Rajauria et al. (2010) found that TFC significantly increased when *H. elongata*, *L. saccharina* and *L. digitata* were heated at 85 °C and 95 °C. However, heating at 121 °C reduced the content almost 3-fold when compared to raw seaweeds [19].

### 2.4. DPPH and ABTS Radical Scavenging Activity and AchE Inhibitory Activity

One of the ways antioxidants inhibit oxidation is through radical scavenging. The assay for evaluating the antioxidant activity in seaweeds utilized a stable free radical, ABTS or DPPH. The decolorization resulting in ABTS and DPPH assay serves as a measure of antioxidant capacity and indicates the presence of electron and/or hydrogen donors in the seaweed extracts.

**Figure 2:**
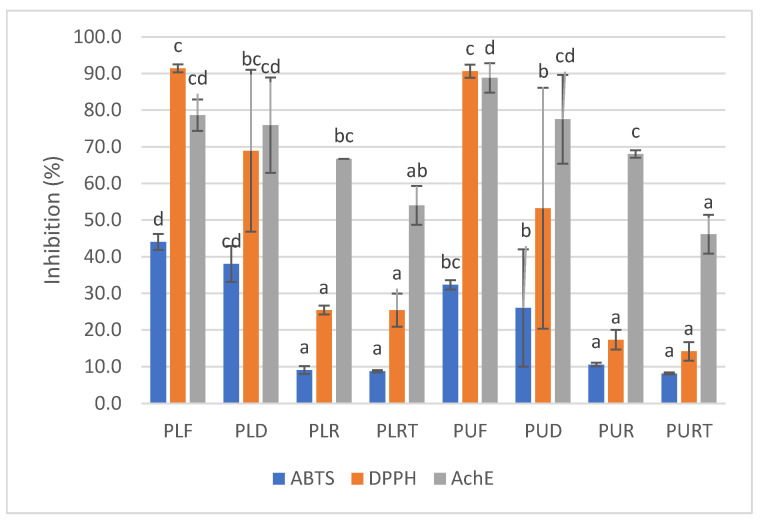
ABTS and DPPH radical scavenging activities and AchE inhibitory activity of fresh (F), dehydrated (D), rehydrated (R) and thermally processed (RT) *P. linearis* (PL) and *P. umbilicalis* (PU) samples. Different letters indicate significant differences (*p* < 0.05) between samples for each activity.

The ABTS radical scavenging activity of fresh and processed seaweeds extracts is shown in Figure 2. The ABTS radical scavenging activity of different *Porphyra* samples was relatively low (8–44%). As can be observed, fresh and dehydrated *P. linearis* samples exhibited the highest ABTS radical scavenging capacity, with inhibition percentages of 44% and 38%, respectively. Furthermore, the ABTS radical scavenging capacity significantly decreased (inhibition percentages between 8 and 11%) in seaweeds that were rehydrated and subjected to thermal treatment, and there were no significant differences between *P. linearis* and *P. umbilicalis.*

In the case of the DPPH radical scavenging activity, fresh samples of *P. linearis* and *P. umbilicalis* exhibited the highest DPPH radical scavenging capacity (with an inhibition percentage of 91%), and dehydration did not cause a significant decrease in this activity. 

Just as in the case of the ABTS radical scavenging activity, the DPPH activity also significantly decreased with the rehydration and thermal treatment (with inhibition percentages between 14 and 25%), and there were no significant differences between *P. linearis* and *P. umbilicalis.*

Gupta et al. (2011) reported comparable findings, indicating that drying at lower temperatures (25 and 30 °C) resulted in a decrease in DPPH radical scavenging activity [16]. Badmus et al. (2019) also observed that elevated temperature treatments of edible brown seaweeds often resulted in products with depleted antioxidant activity [8]. However, several authors have reported that heating could enhance the antioxidant activity of seaweeds with the increase in carotenoids, TPC, and TFC [12,19]. Steam-treated samples of *S. ilicifolium* showed higher antioxidant activity than fresh samples, but in contrast, boiled and sterilized samples had the lowest antioxidant activity [18]. As pointed out by Gupta et al. (2011), the drying process typically leads to a reduction in natural antioxidants in seaweeds [16]. Moreover, extensive or prolonged thermal treatments may result in a substantial loss of natural antioxidants, as most of these compounds are relatively unstable. Li et al. (2006) also reported that a combination of high temperatures and long drying times might destroy some phenolic compounds [20]. Numerous research studies have indicated that the main contributors to the antioxidant activity in seaweeds are phenolic compounds. So, the reduction in ABTS and DPPH radical scavenging activity is often accompanied by a reduction in TPC, as observed in this study. In the present study, a strong significant positive correlation between TPC and DPPH (0.89) and between TPC and ABTS (0.91) was obtained [6].

Acetylcholinesterase is a crucial enzyme responsible for regulating acetylcholine (ACh) levels in the synaptic cleft of neurons, thereby supporting cognitive function. However, the loss or rapid degradation of acetylcholine can result in cholinergic dysfunctions, leading to memory impairment and later to Alzheimer’s disease. Natural inhibitors of cholinesterase have been documented for their inhibitory effects on cholinesterase activity, and these compounds can offer improvement and delay disease progression.

Regarding the anti-Alzheimer’s activity, all samples exhibited a relatively high AchE inhibitory activity (Figure 2), and in general, *P. umbilicalis* samples (46–89%) possessed higher activity than *P. linearis* (54–79%). In addition, rehydration and thermal treatment caused a decrease in AchE inhibitory activity in both species. In the case of *P. umbilicalis*, rehydration and heat treatment caused a decrease of 23 and 40%, but the same did not occur with *P. linearis*. In this seaweed, there were no significant differences between the rehydrated samples and those subjected to heat treatment, and the decrease in this activity compared to fresh samples was approximately 30%.

Research has indicated that phenolic compounds such as phlorotannins, sulphated polysaccharides such as fucoidan or ulvan, and bromophenols have AchE inhibitory activity [21]. However, the AchE inhibitory activity of these compounds can vary based on factors such as seaweed species, extraction methods, and specific chemical structures.

Olasehinde et al. (2019) showed that aqueous ethanol extracts rich in phlorotaninns, phenolic acids, and flavonoids from *Ecklonia maxima*, *Gelidium pristoides*, *Gracilaria gracilis* and *U. lactuca* exhibit AchE inibitory activities [22]. Rengasamy et al. (2015) reported the AchE inhibitory activity of *Codium duthieae*, *Amphiroa beauvoisii*, *Gelidium foliaceum*, *Laurencia complanata*, and *Rhodomelopsis Africana,* with IC_50_ values varying between 0.07 and 0.16 mg/mL [23]. Son et al. (2016) reported that 1mg/mL of methanol extracts of *Ecklonia cava*, *Ecklonia kurome*, and *Myelophycus simplex* exhibited AChE inhibitory activity (with inhibition of 15–35%) [24].

No studies were found in the literature on the effect of rehydration, dehydration, and heat treatment on seaweeds’ AcHE inhibitory activity that would allow for a comparison with the results obtained in this study.

### 2.5. Color Analysis

**Figure 3:**
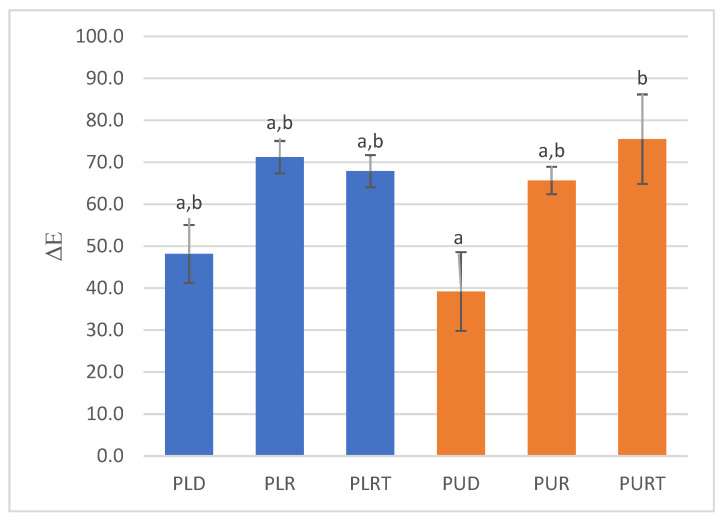
Change in color (ΔE) of dehydrated (D), rehydrated (R) and thermally processed (RT) *P. linearis* (PL) and *P. umbilicalis* (PU) samples. Different letters indicate significant differences (*p* < 0.05) between samples for each activity.

The change in color calculated from color parameters L*, a*, and b* is shown in Figure 3. The smaller values of ΔE indicate that samples are closer in color to fresh seaweeds. As can be seen, the dehydrated samples underwent a significantly lower color change (ΔE_PUD_ = 39.2, ΔE_PLD_ = 48.1). However, rehydration and thermal treatment had a strong effect on color change in the seaweeds (ΔE between 65.7 and 75.5). It was also observed that changes in color were not significantly different between samples subjected to rehydration and thermal treatment. After rehydration, heating to a high temperature (90 °C) had almost no effect on the color of the samples.

The color change in seaweeds after dehydration, rehydration and thermal treatment is related to their composition of pigments. Thus, the changes observed in carotenoid and phycobiliprotein contents after the different treatments may be responsible for the overall color appearance of the seaweeds. Furthermore, exposure of seaweeds to air during processing can also promote oxidative processes which lead to the formation of novel compounds exhibiting different colors or contribute to the fading of current pigments. Maillard reactions can occur during thermal treatment and may produce brown pigments, contributing to a darker color in seaweeds. As can be seen, the rehydrated and thermally processed *Porphyra* samples had lower carotenoid and phycobiliprotein contents (Table 2 and Table 3), which showed that these methods can affect the structure and stability of these pigments, leading to changes in color. However, the samples subjected to these treatments had also lower TPC. In fact, ΔE had a significant high negative correlation with carotenoid content (r^2^ ranging from −0.75 and −0.85) and TPC (r^2^ = −0.89) and with phycobiliprotein content (r^2^ ranging from −0.56 and −0.67).

Rajauria et al. (2010), showed that heat treatment had a strong effect on the change in the color of Irish brown seaweeds. On the other hand, results obtained by Susanto et al. (2016) showed a minimal effect on color change from different heat treatments; however, ΔE was of the same order of magnitude as that obtained in this study for rehydrated and thermally processed *Porphyra* samples [18,19].

## 3. Materials and Methods

### 3.1. Seaweed Species

**Figure 4:**
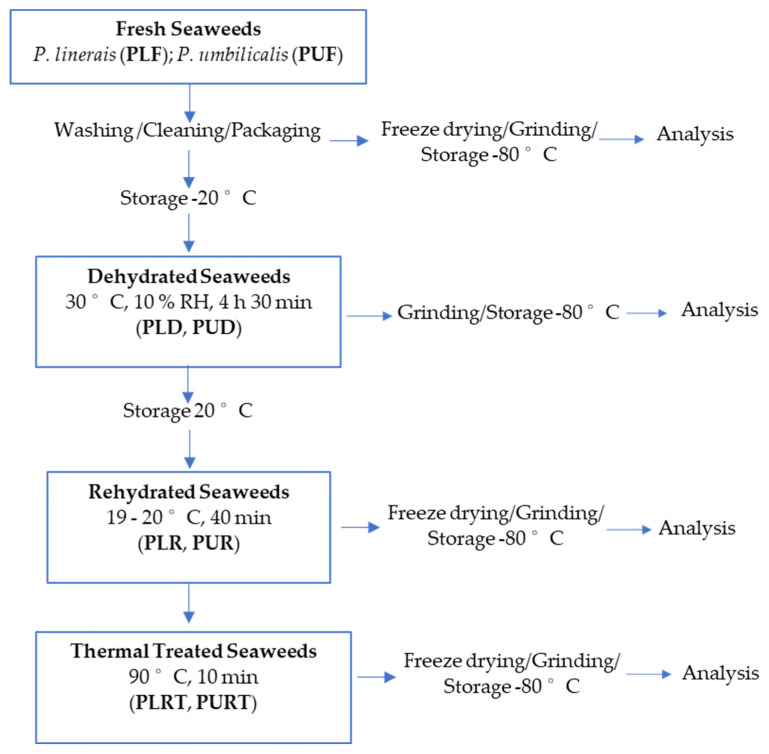
Seaweed treatments and sample codification.

*Porphyra umbilicalis* (Kützing, 1843) and *Porphyra linearis* (Greville, 1830) were harvested in May 2021 at Cabo Mondego (Figueira da Foz, Portugal). After identification, the seaweeds were quickly washed, drained, packed in plastic bags, and transported to the laboratory at Lisbon in refrigerated conditions (within 2 h). At the laboratory, the seaweeds were washed with tap water, simulating the industrial procedure, and drained over nets for approximately 10 min. Then, the seaweeds were packed in propylene bags and divided into two groups: one was frozen at −80 °C following freeze-drying and analysis (characterization of the original properties of fresh macroalgae), and the second one was frozen at −20 °C for following a dehydration process (Figure 4).

### 3.2. Seaweed Treatments

Based on the literature, previous experiments were conducted to test the most appropriate conditions in which to preserve the original quality of red seaweeds, including the bioactive benefits from seaweeds’ consumption. Regarding thermal treatment, the main focus was to test a temperature similar to an industrial and relatively mild heat treatment, such as pasteurization (63–95 °C) but also to test a temperature close to culinary processing, such as boiling (100 °C).

All the treatments (dehydration, rehydration, and thermal treatment) were carried out in duplicate. Table 1 shows the summary of the treatments as well as the samples’ codification.

#### 3.2.1. Dehydration

Frozen seaweeds (−20 °C) were previously thawed at 6 °C in a refrigerator (Fiocchetti, Medika 500, 527 L capacity, Luzzara, Italy) and dehydrated in a CLIMACELL^®^ chamber (Evo Line, 222 L capacity, MM Group, Munich, Germany) with forced air circulation and controlled heating and humidity. The process took place at 30 °C and 10% relative humidity, with air circulation at 0.05 m/s over 4 h 30 min.

Dehydrated seaweeds were packed in polypropylene bags and divided into two parts: one was stored at −80 °C for posterior analysis, and the other part was stored at room temperature (20 °C) and protected from light, for posterior rehydration.

#### 3.2.2. Rehydration

The reconstitution of dehydrated seaweeds was carried out at 19–20 °C using tap water in a proportion of 10 g dehydrated seaweed to 250 mL water over 40 min. Rehydrated seaweeds were immediately divided in two groups: one group was drained for 10 min and subsequently packed in polypropylene bags and stored at −80 °C for posterior freeze-drying and analysis; the second group was immediately thermally treated.

#### 3.2.3. Thermal Treatment

Rehydrated seaweeds were heated in an oven (Rational Combi Master CM6, Landsberg, Germany) at 90 °C for 10 min. Then, after cooling, the seaweeds were packed in polypropylene bags and stored at −80 °C for posterior freeze-drying and analysis.

### 3.3. Seaweeds Analysis

All seaweeds were previously freeze-dried, except the dehydrated ones, milled with a MM400 Mills (Retsch, Haan, Germany), and stored at −80 °C until analysis (Figure 4).

#### 3.3.1. Carotenoid Content

The carotenoid content (neurospene, α-carotene, lutein, zeaxanthin, fucoxanthin, β-carotene and astaxanthin) of seaweeds was measured by the method described by Pires et al. (2017). To extract carotenoids from 1 g of dried seaweed, acetone (5 mL) was added and the mixture was subjected to four extractions for 30 min each. All resulting extracts were combined, and 2.5 mL of water was added. The mixture is then extracted twice with 10 mL of hexane each time. Both hexane extracts were pooled, and 5 mL of a 5% NaCl solution was added. The mixture was subsequently dried using anhydrous Na_2_SO_4_ and the total content of different carotenoids was calculated with the following formula:

(1) $$Carotenoid\;content(\mu g/g\;seaweed)=\frac{Abs\times Vext\times 1000\times MW}{\varepsilon_{1cm}^{1\%}\times 1\times W},$$

where Abs is the absorbance at λ nm of each carotenoid, Vext is the volume of the extract, MW is the molecular weight of each carotenoid, $\varepsilon_{1cm}^{1\%}$ is the coefficient of extinction of each carotenoid, and W is the weight of the sample (g). In Table 3, the wavelength, molecular weight, and extinction coefficients for each carotenoid are presented [25].

**Table 3:** Visible absorption data, molecular weight, and extinction coefficients ($\varepsilon_{1cm}^{1\%}$) for each carotenoid.

Carotenoid	λ (nm)	MM (g mol^−1^)	ε (L mol^−1^ cm^−1^)	Reference
Neurosporene	440	538.90	157,000	[26]
α-Carotene	445	536.88	145,000	[26]
Lutein	450	568.87	145,000	[27]
Zeaxanthin	450	568.88	134,000	[26]
Fucoxanthin	453	658.92	109,000	[28]
β-Carotene	453	536.88	139,000	[28]
Astaxanthin	470	596.85	125,000	[26]

All experiments were performed in triplicate.

#### 3.3.2. Phycobiliprotein Content

Phycobiliprotein extraction from dried seaweeds was performed using the method described by Pan-utai and Iamtham (2019) with slight modifications [29]. One hundred milligrams of dried seaweeds were homogenized at 15,000 rpm for 1 min (Polytron, KINEMATICA, Malters, Switzerland) with 10 mL of 0.01 M sodium phosphate buffer (pH 7.0). The resulting mixture was incubated with agitation at 50 °C for 24 h in the dark. Afterwards, the mixture was centrifuged (6000 rpm, 10 min, 4 °C) to separate crude phycobiliproteins, and the volume of supernatant was adjusted to 10 mL. The absorbance of sample solutions was recorded between 400 and 700 nm at a scan speed of 500 nm/min in an UV–vis spectrophotometer (Evolution 201, Thermo Scientific, Waltham, MA, USA). The absorbance values for the wavelengths of maximum absorption of the phycoerythrin (λ = 575 nm), phycoerythrocyanin (λ = 560 nm), phycocyanin (λ = 620 nm), allophycocyanin (λ = 560 nm), and allophycocyanin-β (λ = 675 nm) were determined. All experiments were performed in triplicate.

#### 3.3.3. Seaweed Extracts

The seaweed extraction procedure for the determination of total phenolic and flavonoid contents and antioxidant and acetylcholinesterase inhibitory activities was performed according to the method described by Monteiro et al. (2020) with minor modifications. Briefly, 500 mg of freeze-dried seaweed samples (fresh, dehydrated, rehydrated. and thermally treated) were mixed with 10 mL of 80% methanol, homogenized, and incubated for 15 min with continuous agitation in the dark at room temperature. The mixture was centrifuged for 15 min at 10,600× *g* at 4 °C. This procedure was repeated three times, supernatants from successive extractions were pooled, and final volume was adjusted to 50 mL with 80% methanol. The extracts were filtered through 0.22 µm filters and stored at 4 °C in the dark before analysis [30].

#### 3.3.4. Total Phenolic Content (TPC)

The determination of TPC was carried out using the Folin–Ciocalteu colorimetric method, where gallic acid was used as standard (5–500 mg/mL). Briefly, 150 μL of seaweed extract solution (3–5 mg/mL) was mixed with 750 μL of Folin–Ciocalteu reagent that was diluted in a 1:10 ratio. Following a 4 min incubation period at room temperature, 600 μL of 10% sodium carbonate was added to the mixture. The absorbance of the resulting mixture was then measured at a wavelength of 765 nm after it had been allowed to incubate at room temperature in the dark for 2 h. In the blank, distilled water was used instead of seaweed extract solution. The results obtained were expressed as mg of gallic acid equivalent (GAE) per g of seaweed extract dry weight. All analyses were carried out at least three times, and the results are presented as a mean value ± standard deviation (SD) [31].

#### 3.3.5. Total Flavonoid Content (TFC)

The determination of TFC was performed using the spectrophotometric method, as outlined by Pekal et al. (2014). In brief, 1 mL of the sample solution (1–5 mg/mL) was mixed with 0.5 mL of 2% AlCl_3_, and then 0.5 mL of water was added. The mixture was thoroughly shaken and then allowed to incubate at room temperature for 10 min. After this, 4 mL of water was added, and the absorbance was measured at a wavelength of 425 nm. For the blank, water was used in place of AlCl_3_ solution. Quercetin (25–250 μM) was used as the standard, and the results were expressed as mg of quercetin equivalent (QE) per gram of seaweed extract’s dry weight. All analyses were carried out at least three times, and the results were presented as a mean value ± standard deviation (SD) [32].

#### 3.3.6. Antioxidant Activity

##### DPPH Radical Scavenging Activity

The determination of DPPH radical scavenging activity was performed according to the method of Sapatinha et al. (2021). Briefly, 100 µL of the different seaweed extracts (1–20 mg/mL) was added and mixed with 1.0 mL of 0.1 mM DPPH solution in 95% ethanol. The mixture was vortexed and then placed in a water bath at 30 °C for 30 min in the dark. Thereafter, samples were centrifuged at 10,000× *g* for 5 min and the absorbance was measured at 517 nm using an UV–visible spectrophotometer (Evolution 201, Thermo Fisher Scientific, Waltham, MA, USA). The control was prepared in the same way, but distilled water was used instead of the sample solution. The radical scavenging activity of DPPH was calculated by the percentage inhibition of DPPH, as follows:

(2) $$\mathrm{DPPH}\;\mathrm{scaveging}\;\mathrm{activity}\;(\%)=\frac{A_{C}-{\;A}_{S}}{A_{C}}\times 100,$$

where A_S_ and A_C_ correspond to the absorbance of sample and control, respectively. All analyses were made at least in triplicate, and the results are presented as mean values. The EC50 value was calculated for each seaweed extract [31].

##### ABTS Radical Scavenging Activity

The ABTS radical scavenging activity of seaweed extracts was performed according to Sapatinha et al., 2021. The ABTS radical cation ABTS^•+^ was prepared with a final concentration of 7 mM ABTS in 2.45 mM potassium persulfate. This mixture was kept in the dark at room temperature for 16 h before use. ABTS^•+^ solution was diluted with 5 mM sodium phosphate buffer (pH 7.4) to obtain an absorbance value of 0.70 ± 0.02 at 734 nm. A 20 µL of solution at different seaweed extract concentrations (0.5–20 mg/mL) was mixed with 2 mL of ABTS^•+^ solution and then incubated in the dark at 30 °C (water bath) for 6 min. The absorbance values of the mixture were measured at 734 nm using a UV–visible spectrophotometer (Evolution 201, Thermo Fisher Scientific, Waltham, MA, USA). The control was prepared in the same manner, using distilled water instead of the sample solution. All determinations were made at least in triplicate, and the EC50 value was calculated for each seaweed. The ABTS radical scavenging activity was calculated according to the following equation:

(3) $${\mathrm{ABTS}}^{•+}\mathrm{scavenging}\;\mathrm{activity}\;(\%)=\frac{A_{C}-{\;A}_{S}}{A_{C}}\times 100,$$

where AC represents the absorbance of the control and AS represents the absorbance of sample [31].

##### Acetylcholinesterase (AChE) Inhibitory Activity

The AChE inhibitory activity was evaluated by the spectrophotometric method developed by Ahn et al. (2010) using acetylcholine (ACh) as a substrate. The reaction mixture (containing 80 mL of 100 mM sodium phosphate buffer (pH 8.0)), 40 mL of sample solution, and 40 mL of AChE (0.36 UmL^−1^) were mixed and incubated for 15 min at 37 °C. The reactions were then initiated via the addition of 20 mL of DTNB (0.5 mM) and 20 mL of ACh (0.6 mM). The hydrolysis of ACh was monitored by the following formation of yellow 5-thio-2-nitro-benzoate anion at 400 nm for 5 min, which resulted from the reaction of DTNB with thiocholine, released by the enzymatic hydrolysis of ACh. The percentage inhibition of AChE was calculated by the following formula:

(4) $$\mathrm{AChE}\;\mathrm{inhibit}\mathrm{ory}\;\mathrm{activity}\;(\%)=\frac{A_{B}-{\;A}_{S}}{A_{B}}\times 100$$

where A_B_ is the absorbance of the 5-thio-2-nitro-benzoate anion in the reaction with water instead of the sample, and A_S_ is the absorbance of the 5-thio-2-nitro-benzoate anion in the reaction with the sample [33].

#### 3.3.7. Color Analysis

The color of seaweed samples was measured using a colorimeter CR-300 (Konica Minolta Inc., Tokyo, Japan) with standard illuminant D65. Tristimulus color coordinates (CIELAB-system) were used to measure the degree of lightness (L*), redness (a*), and yellowness (b*). In order to evaluate the effect of dehydration, rehydration, and heat treatment on seaweed color, the total color difference was calculated using the following equation:

ΔE = [(L* − L_0_*)^2^ + (a* − a_0_*)^2^ + (b* − b_0_*)^2^]^1/2^, (5)

ΔE quantifies the overall color difference of a sample (L*, a*, b*) when compared to a reference sample (L_0_*, a_0_*, b_0_*) [34]. In this study, fresh seaweeds (PLF and PUF) were used as reference samples.

### 3.4. Statistical Analysis

The results of the analyses were reported as mean values ± standard deviation (SD). All statistical analyses were performed using the software STATISTICA© version 12 from StatSoft, Inc. (Tulsa, OK, USA). Differences among mean values of the groups were tested using a one-way analysis of variance (ANOVA), followed by the Tukey test. Pearson correlation analyses between several variables (e.g., DPPH, TPC and ABTS) were also performed. Statistical significance was considered at *p* < 0.05.

## 4. Conclusions

The results of this study indicate that dehydration (30 °C, 10% RH, 4 h 30 min), rehydration (at 19–20 °C, 40 min), and thermal treatment (90 °C, 10 min) influence the color, the content of bioactive compounds, and the antioxidant and anti-Alzheimer’s activities of *P. linearis* and *P. umbilicalis*. The application of thermal treatment resulted in the most notable decrease in bioactive compounds and antioxidant activity, with differences observed between the two species. However, AchE inhibitory activity remained relatively high in all treated seaweeds. Changes in color (ΔE) were significant, especially after rehydration and thermal treatment, with this effect being more pronounced in *P. umbilicalis*. This change in color resulted from a reduction in carotenoid and phycobiliprotein contents. Overall, *P. umbilicalis* exhibited higher bioactive compound levels, and consequently, higher antioxidant and AchE inhibitory activity.

As drying/dehydrating stands as the primary method for seaweed commercialization, both dehydrated species exhibit significant potential for various applications. These include their use in juices and soups (preferably pasteurized or subjected to high hydrostatic pressure), in stews, and as ingredients for food supplements (provided they are processed at low temperatures). This presents us with an opportunity to produce sustainable food commodities enriched with advantageous bioactive properties.

## Figures and Tables

**Table 1:** Neurosporene, α-carotene, lutein, zeaxanthin, fucoxanthin, β-carotene and astaxanthin content (μg/g dw) of fresh (F), dehydrated (D), rehydrated (R), and thermally processed (RT) *P. linearis* (PL) and *P. umbilicalis* (PU) samples. Different letters in each column indicate significant differences (*p* < 0.05) between samples.

Sample	Neurosporene	α-Carotene	Lutein	Zeaxanthin	Fucoxanthin	β-Carotene	Astaxanthin
PLF	1309.8 ± 73.8 ^b^	1168.5 ± 73.3 ^b^	1081.3 ± 64.4 ^c^	1170.0 ± 69.6 ^a^	1525.8 ± 92.3 ^a^	974.9 ± 59.0 ^a^	1186.1 ± 67.5 ^a^
PLD	978.3 ± 71.8 ^ab^	844.9 ± 149.8 ^ab^	682.7 ± 42.3 ^abc^	847.4 ± 153.7 ^a^	1317.8 ± 485.6 ^a^	746.2 ± 174.8 ^a^	838.5 ± 156.7 ^a^
PLR	559.5 ± 119.4 ^ab^	493.9 ± 97.4 ^ab^	456.7 ± 88.8 ^ab^	494.2 ± 96.1 ^a^	645.6 ± 126.5 ^a^	412.5 ± 80.8 ^a^	490.8 ± 94.5 ^a^
PLRT	700.2 ± 283.5 ^ab^	696.8 ± 250.3 ^ab^	677.2 ± 18.9 ^abc^	732.8 ± 257.0 ^a^	979.3 ± 581.3 ^a^	625.7 ± 255.6 ^a^	756.3 ± 260.7 ^a^
PUF	1224.7 ± 54.4 ^ab^	1097.5 ± 56.6 ^ab^	1020.8 ± 58.8 ^bc^	1104.6 ± 63.4 ^a^	1441.2 ± 82.1 ^a^	920.8 ± 52.4 ^a^	1116.3 ± 66.2 ^a^
PUR	695.4 ± 373.8 ^ab^	608.0 ± 336.2 ^ab^	564.2 ± 316.3 ^abc^	610.5 ± 342.2 ^a^	807.6 ± 448.0 ^a^	516.0 ± 286.2 ^a^	609.3 ± 345.7 ^a^
PUD	742.8 ± 283.5 ^ab^	633.1 ± 250.3 ^ab^	419.0 ± 81.2 ^a^	635.1 ± 257.0 ^a^	1008.3 ± 581.3 ^a^	562.4 ± 255.6 ^a^	627.4 ± 260.7 ^a^
PURT	439.4 ± 256.3 ^a^	427.3 ± 250.3 ^a^	409.6 ± 241.9 ^a^	443.2 ± 261.8 ^a^	590.0 ± 345.4 ^a^	377.0 ± 220.7 ^a^	454.6 ± 267.4 ^a^

**Table 2:** Phycoerythrin, phycoeythrocyanin, phycocyanin, allophycocyanin, and allophycocyanin-β (AU) of fresh (F), dehydrated (D), rehydrated (R), and thermally processed (RT) *P. linearis* (PL) and *P. umbilicalis* (PU) samples. Different letters in each column indicate significant differences (*p* < 0.05) between samples.

Sample	Phycoerythrin	Phycoerythrocyanin	Phycocyanin	Allophycocyanin	Allophycocyanin-β
PLF	1.16 ± 0.01 ^b^	0.70 ± 0.01 ^ab^	0.71 ± 0.00 ^c^	0.31 ± 0.01 ^abc^	0.10 ± 0.02 ^a^
PLD	0.97 ± 0.22 ^ab^	0.99 ± 0.31 ^b^	0.79 ± 0.01 ^c^	0.48 ± 0.03 ^c^	0.57 ± 0.16 ^a^
PLR	1.04 ± 0.41 ^b^	0.61 ± 0.28 ^ab^	0.62 ± 0.29 ^bc^	0.25 ± 0.13 ^abc^	0.11 ± 0.10 ^a^
PLRT	0.23 ± 0.04 ^a^	0.21 ± 0.04 ^a^	0.19 ± 0.04 ^ab^	0.18 ± 0.05 ^ab^	0.20 ± 0.05 ^a^
PUF	1.33 ± 0.08 ^b^	0.82 ± 0.07 ^b^	0.82 ± 0.09 ^c^	0.41 ± 0.09 ^bc^	0.38 ± 0.25 ^a^
PUD	0.90 ± 0.31 ^ab^	0.88 ± 0.18 ^b^	0.73 ± 0.06 ^c^	0.43 ± 0.00 ^c^	0.36 ± 0.30 ^a^
PUR	0.78 ± 0.11 ^ab^	0.46 ± 0.07 ^ab^	0.50 ± 0.05 ^abc^	0.18 ± 0.05 ^ab^	0.06 ± 0.03 ^a^
PURT	0.21 ± 0.03 ^a^	0.19 ± 0.02 ^a^	0.16 ± 0.02 ^a^	0.14 ± 0.02 ^a^	0.18 ± 0.02 ^a^

## Data Availability

The data presented in this study are available on request from the corresponding author because it is obtained within the scope of a national project.
